# Synergistic effect of low‐frequency ultrasound and antibiotics on the treatment of *Klebsiella pneumoniae* pneumonia in mice

**DOI:** 10.1111/1751-7915.14134

**Published:** 2022-08-24

**Authors:** Kaicheng Yan, Tianli Yang, Juan Xu, Liuhan Dong, Jin Wang, Yun Cai

**Affiliations:** ^1^ Medical School of Chinese PLA Beijing China; ^2^ Department of Pharmacy, Center of Medicine Clinical Research, Medical Supplies Center Chinese PLA General Hospital Beijing China

## Abstract

The antibiotic‐resistant *Klebsiella pneumoniae* (Kp) has become a significant crisis in treating pneumonia. Low‐frequency ultrasound (LFU) is promising to overcome the obstacles. Mice were infected with bioluminescent Kp Xen39 by intratracheal injection to study the therapeutic effect of LFU in combination with antibiotics. The counts per second (CPS) were assessed with an animal biophoton imaging system. Bacterial clearance, histopathology, and the concentrations of cytokines were determined to evaluate the therapeutic effect. LC–MS/MS was used to detect the distribution of antibiotics in the lung and plasma. LFU in combination with meropenem (MEM) or amikacin (AMK) significantly improved the behavioural state of mice. The CPS of the LFU combination group were more significantly decreased compared with those of the antibiotic alone groups. The average colony‐forming units of lung tissue in the LFU combination groups were also lower than those of the antibiotic groups. Although no significant changes of cytokines (IL‐6 and TNF‐α) in plasma and bronchoalveolar lavage fluid were observed, LFU in combination with antibiotics showed less inflammatory damage from histopathological results compared with the antibiotic‐alone groups. Moreover, 10 min of LFU treatment promoted the distribution of MEM and AMK in mouse lung tissue at 60 and 30 min, respectively, after dosage. LFU could enhance the effectiveness of MEM and AMK in the treatment of Kp‐induced pneumonia, which might be attributed to the fact that LFU could promote the distribution of antibiotics in lung tissue and reduce inflammatory injury.

## Graphical abstract

In the present *in vivo* study, we provide evidence showing that LFU can improve the efficacy of meropenem (MEM) or amikacin (AMK) in Kp pneumonia model. LFU combined with antibiotics significantly improved the survival state of mice, decrease the counts per second (CPS), accelerated the clearance of bacteria and reduced inflammatory injury of lung. The application of LFU in the treatment of pulmonary infection may have far‐reaching significance.

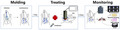

## INTRODUCTION

As a type of Gram‐negative bacteria, *Klebsiella pneumoniae* (Kp) exists widely in the environment. In China, the isolation rate of Kp in respiratory specimens has exceeded that of *Acinetobacter baumannii*, rising to first place since 2017 (Hu et al., 2019). Kp has become the main cause of medical‐related infection in hospitals and the risk factor of serious community‐acquired infection (Holt et al., 2015). Hospital‐acquired pneumonia caused by Kp is becoming more common worldwide, which is associated with high morbidity and mortality (Zhang et al., 2020). Crucially, antibiotic‐resistant Kp is emerging, especially carbapenem‐resistant Kp, which has been identified as an urgent threat to human health (Navon‐Venezia et al., 2017). A recent study has found that carbapenem‐resistant Kp accounts for 90% of the total isolated strains in a multicenter clinical study from different global regions (Wang et al., 2021). For the empirical treatment of KP infection with antibiotics, cephalosporins, carbapenems, and quinolones are usually selected and β‐lactamase inhibitor mixture, tigecycline, or colistin is selected for KP producing extended‐spectrum β‐lactamase (ESBL) or carbapenem‐resistant strains (Chen & Xiang, 2018; van Duin et al., 2018). The endless emergence of multidrug‐resistant (MDR) bacteria calls for production of new antibiotics. Although some new antibiotics have been developed in recent years, the emergence of MDR bacteria is closely followed. Therefore, it is necessary to find some new adjuvants to overcome the treatment obstacles of pneumonia caused by Kp.

Low‐frequency ultrasound (LFU) refers to a type of ultrasound with a lower frequency, generally ranging from 20 kHz to 1 MHz (Lentacker et al., 2010). Compared with high‐frequency ultrasound, LFU is characterized by lower frequency, longer wavelength, relatively less sound energy absorption, and higher power, making LFU easier to penetrate body tissue and causing less damage (Gao et al., 2014). LFU has a variety of biological effects, such as mechanical effects, thermal effects, and cavitation effects (Xie et al., 2008). More and more attention has been paid to the role of LFU in clinical treatment, such as promoting percutaneous penetration of medicines, accelerating coronary plaque ablation and thrombolysis, assisting in inhibiting tumour formation, and alleviating diabetic peripheral neuropathic pain (Auboire et al., 2018; Polat et al., 2011; Puts et al., 2018).

With the treatment of conventional antibiotics, physical LFU can not only enhance the anti‐infection ability of antibiotics but also reduce the risk of bacterial antibiotic resistance (Conner‐Kerr et al., 2010). We and other researchers have found that LFU can improve the antibacterial effect of a variety of antibiotics on planktonic and biofilm bacteria, such as *Pseudomonas aeruginosa* (PA), Methicillin‐resistant *Staphylococcus aureus* (MRSA), and Kp (Liu et al., 2016; Pitt et al., 1994; Wang et al., 2018). However, most of these reports are *in vitro* studies, and there is no exploration of pulmonary infection. In the present study, we aimed to investigate the *in vivo* synergistic effect of LFU in combination with antibiotics on the pulmonary infection through a mouse model of pneumonia. Moreover, the possible underlying mechanisms were further explored.

## EXPERIMENTAL PROCEDURES

### Strains, antibiotics, and antimicrobial susceptibility test

A bioluminescent strain of Kp Xen39 was purchased from PerkinElmer, which carries *lux* CDABE gene operon and exhibits autofluorescence. *Escherichia coli* ATCC25922 was used as a quality control (QC) strain when measuring the minimum inhibitory concentration (MIC) of Kp Xen39 (Humphries et al., 2019). Meropenem (MEM) and tigecycline were supplied by Sigma. Amikacin (AMK) and levofloxacin were provided by the China National Institute for the Control of Pharmaceutical and Biological Products. To test the susceptibility of routinely used antibiotics against KP in the clinic, we selected the classical MEM (carbapenem), AMK (aminoglycoside), levofloxacin (quinolone), and tigecycline (tetracycline). According to Clinical and Laboratory Standards Institute (CLSI) guidelines, the broth microdilution method was used for the susceptibility test of four antibiotics (CLSI, 2021). The QC bacteria and the bacteria to be tested were cultured at the same time. If the MIC of the QC bacteria was within the normal range, then the results of this drug susceptibility test were reliable. Briefly, the concentrations of antibiotics in 96‐well plates ranged from 0 to 1,024 μg/ml. After the high‐concentration mother liquor of the four antibiotics was prepared, the double dilution method was used. The MIC determination of KP for different antibiotics was repeated three times. The Kp Xen39 and *Escherichia coli* ATCC25922 were grown on Mueller–Hinton agar (MHA, BD Difco), and then representative colonies were selected and suspended in Mueller–Hinton broth (MHB) at 37°C for 8 h. The bacterial solution was turbidized to 0.5 McFarland units (1 × 10^8^ colony‐forming units, CFU/ml) using a turbidimeter. After 100‐fold dilution, 100 μl of the resultant bacterial suspension (1 × 10^6^ CFU/ml) was loaded into each well of a 96‐well plate, followed by incubation at 37°C for 24 h.

### LFU apparatus

The LFU apparatus was provided by Beijing Nava Medical Technology. The fixed working frequency was 29.36 kHz, and the effective output intensity was 250–300 mW/cm^2^. In the present study, the LFU was operated at 270 mW/cm^2^ for 10 min, which was determined according to the degree of burn of mouse skin under different output power and action time in our preliminary experiment. The effective working diameter of the ultrasonic tool head was 1.5 cm, and the working mode was pulse modulation. The mice were placed in the anaesthesia induction box for preliminary anaesthesia, and then the mouth and nose of the mice were placed in the anaesthesia mask. Under the continuous inhalation of isoflurane, the mice were placed in the supine position, the limbs of the mice were extended, the chest skin of the mice was fully exposed, and the limbs of the mice were fixed on the multifunctional small animal experimental console with an adjustable angle with medical tape. An appropriate amount of ultrasonic couplant was applied to the chest skin of mice, and the working surface of the LFU probe was adjusted to fully fit the chest of mice without gap and without compressing the chest of mice.

### Animals and pneumonia model

**FIGURE 1:**
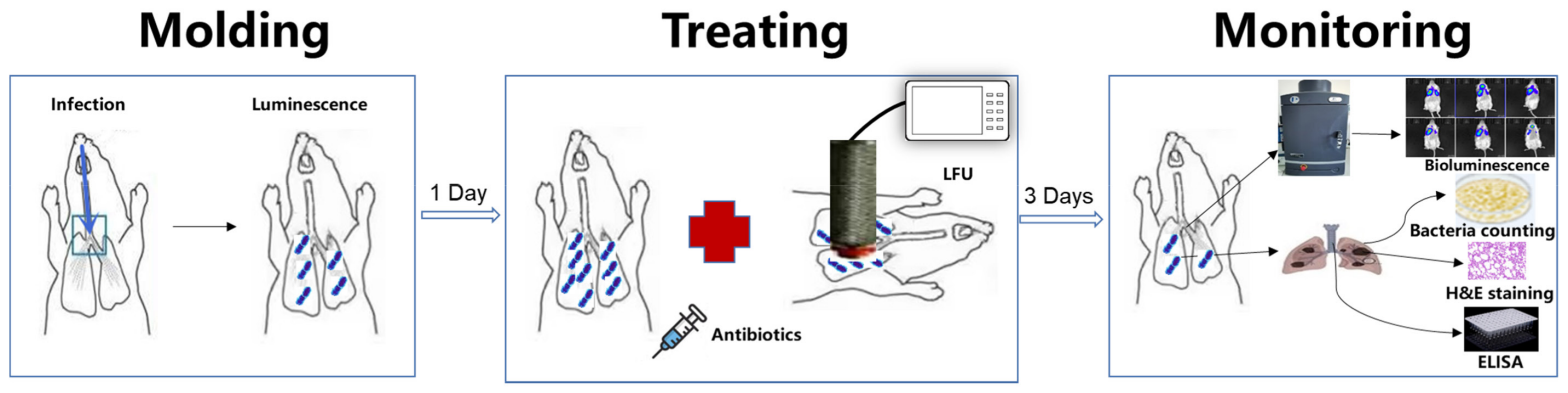
Schematic diagram of the mouse experiment process.

ICR male mice, 8–10 weeks old, weighing 27–32 g (Animal Facility, PLA General Hospital, China) were used in the present study. All animal experiments and procedures were approved by the animal ethics committee of the Chinese PLA General Hospital (SQ2020095). Mice were maintained in a pathogen‐free environment with an ambient temperature of 21–23°C and a 12‐h dark–light cycle. Mice were housed in sterile cages with sterile feed and water in the Animal Experiment Center of PLA General Hospital. Cyclophosphamide (Sigma‐Aldrich) was injected intraperitoneally (ip) 4 days (150 mg/kg body weight) and 1 day (100 mg/kg body weight) before the experiment to reduce neutrophil content in mice (Hu et al., 2020). The lung infection was established as previously described (Yamashita et al., 2019). Briefly, neutropenic ICR mice were anaesthetised (pentobarbital, ip) before inoculation of 100 μl of Kp Xen39 strain suspension (3 × 10^8^ CFU/ml) via intratracheal instillation. The injected bacterial concentration and dosage were determined based on our previously published research about establishing a pneumonia infection model (Hu et al., 2020). The animals were held vertically for 1 min after inoculation and then placed in the cage in a supine position to recover. MEM, AMK, levofloxacin, and tigecycline can all be used for KP infection. The ratio of antibiotic concentration in lung tissue to plasma in 1 h is summarized in Table 1 according to published data. To better observe the effect of LFU on the distribution of antibiotics in lung tissue, MEM and AMK with low distribution were selected as the research antibiotics (Najmeddin et al., 2020; Wiseman et al., 1995). Since the concentrations of tigecycline and levofloxacin distributed in lung tissue are higher than those in plasma (De Pascale et al., 2020; Fish & Chow, 1997), they were not evaluated in the following study. The mice were divided into seven groups with 10 mice in each group as follows: negative control group, infection group, LFU group, MEM group, MEM + LFU group, AMK group, and AMK + LFU group. Except that the negative control group was inoculated with saline, the other groups were inoculated with Kp Xen39 suspension. MEM (75 mg/kg, q.8 h, ip) or AMK (67.5 mg/kg, q.12 h, ip) was used 24 h after infection for 3 days. A dose of 75 mg/kg for MEM and 67.5 mg/kg for AMK corresponds to the recommended dosage for humans, which are 500 mg/body for MEM and 7.5 mg/kg for AMK (Aye et al., 2020; Bartoloni et al., 1999). LFU was applied immediately for 10 min after each antibiotic administration. A schematic diagram of the mouse experiment process was added in Figure 1 to explain the experimental procedure better. The infection and LFU groups were treated at 24 h after infection and intraperitoneally injected with the same amount of normal saline (NS) as antibiotics. The mice in the LFU group were treated with LFU for 10 min after each injection of NS, three times a day for 3 consecutive days. In the infected group, LFU was not performed.

**Table 1:** MICs of antimicrobial agents against Kp Xen39 and the ratio of antibiotic concentration in lung/plasma at 1 h after dosage.

Antimicrobial agents	MIC (μg/ml)		MIC (interpretive criterion (μg/ml))			Quality control standard	Concentration ratio (%)
	Kp Xen39	ATCC 25922	Susceptible	Intermediate	Resistant	ATCC 25922	Lung: plasma (1 h)
MEM	0.03125	0.0156	≤1	2	≥4	0.008–0.06	40 (Wiseman et al., 1995)
Tigecycline	0.5	0.0625	≤4	8	≥16	0.03–0.25	153 (De Pascale et al., 2020)
AMK	4	4	≤16	32	≥64	0.5–4	10 (Najmeddin et al., 2020)
Levofloxacin	0.25	0.0156	≤0.5	1	≥2	0.008–0.06	200 (Fish & Chow, 1997)

### Bioluminescence *in vivo*

All mice were imaged on an IVIS Lumina K Series III living animal biophoton imaging system (PerkinElmer) immediately after infection. Signals were collected from the defined region of interest (ROI) using the contour ROI tool, and the average flux intensity (p/s/cm^2^/sr) was analysed using Living Image Software 4.7.3. Each group of mice was gently anaesthetised with isoflurane through a small animal anaesthesia machine (RWD‐R540). At 24 h after infection, the infected mice in each group were treated according to the dosage of antibiotics or (and) LFU for 3 days, and bioluminescence imaging was performed on all mice every day to quantify the bacterial load.

### Lung bacterial clearance assay

Mice in each group were sacrificed at 96 h after inoculation (72 h after treatment). The blood and bronchoalveolar lavage fluid (BALF) samples were collected, and the right lungs of the mice were dissected and homogenized in sterile PBS (1 ml: 100 mg). Serial dilutions (1:10) were prepared in PBS and inoculated on MHA plates, followed by incubation at 37°C for 24 h. Subsequently, the bacterial colonies were counted, and the colony count formation of the lung homogenate was calculated and expressed as CFU/g.

### Histological sample preparation and histopathological injury scores

The left lung of mice was fixed in 10% neutral buffered formalin for 24 h, embedded in paraffin, and cut into 5—μm‐thick sections. The sections were stained with haematoxylin/eosin (H&E) and observed under an optical microscope. To generate a lung injury score, a total of 300 alveoli on each slide were counted at a magnification of 400. Within each area, scores were assigned according to predetermined criteria. All points of each category were added and weighted according to their relative importance. Lung injury was scored according to four aspects (Matute‐Bello et al., 2001): (1) alveolar septal congestion; (2) bleeding; (3) aggregation or infiltration of neutrophils into the alveoli; and (4) fibrin chains in the alveoli. Each item was scored on a four‐point scale: 3—maximum injury; 2—moderate injury; 1—slight damage; and 0—minimum (minor) damage. The damage score was calculated according to the following formula: injury score = [(alveolar haemorrhage points/no. of fields) + 2 × (alveolar infiltrate points/no. of fields) + 3 × (fibrin points/no. of fields) + (alveolar septal congestion/no. of fields)]/total number of alveoli counted.

### Cytokine measurement in plasma and BALF

After 3 days of treatment, blood samples were obtained by eye extraction, and BALF was acquired by intratracheal injection of 1 ml sterilized PBS into the lungs and immediate vacuum suction. These samples were centrifuged at 3500 r/min for 10 min at 4°C. The supernatant was collected and frozen at −80°C until for enzyme‐linked immunosorbent assay (ELISA). The concentrations of IL‐6 and TNF‐α in plasma and BALF were determined by specific mouse ELISA (MlBio, Shanghai, China) according to the manufacturer's instructions. For data analysis, the curve fitting was applied to the standard, and the sample concentration was inferred from the standard curve using four‐parameter logic software.

### Determination of antibiotics in plasma and lung homogenate by LC–MS/MS

A total of 132 healthy ICR mice in four groups were injected with MEM (75 mg/kg) or AMK (67.5 mg/kg) according to body weight *via* the tail vein. Two groups were treated with antibiotics alone, and the other two groups were administered with LFU in combination with corresponding antibiotics. LFU was used immediately after each antibiotic administration for 10 min. Plasma and lung tissue homogenates were taken at 5, 10, 20, 30, 45, and 60 min after administration in the antibiotic groups and at 10, 20, 30, 45, and 60 min in the LFU combination groups. The experimental groups were designed according to the peak time of MEM and AMK plasma concentrations and the lower limit of quantitation (LLOQ) (Marsot et al., 2017; Takata et al., 2004). After 1 h, the drug concentration in the mouse lung tissue was extremely low (below LLOQ). There were six mice in each group at each time point. The concentrations of MEM and AMK in plasma and lung homogenate were determined by high‐performance liquid chromatography coupled with mass spectrometry (LC–MS/MS) (Agilent Technologies 3000). All sample processing and thawing of frozen plasma samples were performed at 4°C. The specific detection methods of MEM and AMK are shown in Table 2. After verification, these methods were stable and effective.

**Table 2:** Chromatographic and mass spectrometric conditions, preparation of the standard solution, and pretreatment of MEM and AMK.

Methods		MEM	AMK
Chromatographic conditions	Chromatographic column	Eclipse Plus C18 (2.1 × 100 mm, 3.5 μm)	Porosehell 120 HILIIC‐Z (2.1 × 100 mm, 2.7 μm)
	Mobile phase	A: water (0.1% formic acid); B: acetonitrile	A: ammonium acetate (1% formic acid); B: acetonitrile
	Elution gradient	0–3 min: 95% A, 5% B; 3–3.1 min: 30% A, 70% B; 3.1–5.2 min: 0% A, 100% B; 5.2–7 min: 95% A, 5% B; 7–10 min: 95% A, 5% B	0–7 min: 70% A, 30% B
	Current speed	0.4 ml/min	0.2 ml/min
	Column temperature	35	40
	Injection volume	5 μl	10 μl
	Internal standard	Fluconazol	Fluconazol
Mass‐spectrum conditions	Ionization mode	Electrospray ionization source	Electrospray ionization source
	Measured object ion pair	*m*/*z* ~ 384.2/144.1	*m*/*z* ~ 586.2/425.2
	Internal standard ion pair	*m*/*z* ~ 307.0/238.0	*m*/*z* ~ 307.0/238.0
Solution preparation	Standard	500, 200, 100, 50, 10, 5, 2.5, and 1 μg/ml	500, 200, 100, 80, 50, 20, and 5 μg/ml
	Quality control	200, 20, and 2 μg/ml	150, 80, and 10 μg/ml
	Internal standard	1 μg/ml	1 μg/ml
Sample handling	Preparation of medicated plasma (homogenate)	50 μl MEM + 450 μl plasma (homogenate)	50 μl AMK + 450 μl plasma (homogenate)
	Pretreatment	50 μl medicated plasma (homogenate) + 200 μl acetonitrile (fluconazole)	150 μl medicated plasma (homogenate) + 50 μl fluconazole+400 μl methanol
Retention time	Target object	2.70 min	1.00 min
	Internal standard	3.24 min	2.46 min

### Statistical analysis

Data were expressed as mean ± standard deviation (SD). Ordinary one‐way ANOVA was used to compare counts per second (CPS), CFU, histopathological scores, and cytokine concentrations. Holmes–Sidak corrected multiple t‐test was used to evaluate antibiotic concentrations in plasma and lung homogenates. GraphPad Prism software version 8.0 (GraphPad Software) was used for statistical analysis. A *p* < 0.05 was considered statistically significant.

## RESULTS

### MICs for antimicrobial agents

Both Kp Xen39 and *Escherichia coli* ATCC25922 are susceptible strains. Table 1 summarizes the MICs of various antimicrobial agents. The Kp Xen39 and ATCC 25922 strains were both susceptible to MEM, tigecycline, levofloxacin, and AMK.

### Behavioural changes of mice before and after treatment

Before the Kp Xen39 infection, all mice had smooth and shiny hair, as well as a normal diet, and they were lively, active, and sensitive to various external stimuli, such as sound and light. At 24 h after inoculation, mice had different degrees of dry and matte hair, increased cheese‐like secretion from the corners of the eyes, shortness of breath, dyspnea, decreased diet, weight loss, mental depression, curled up laziness, and obvious slow response to the outside world. At 96 h, the infection‐related symptoms of mice in the infection group and LFU group were the most serious. Most mice had more secretions at the corners of their eyes, more like cheese secretions. The eyelids of mice were closed, and they did not drink water most of the time. They seemed to be in a state of continuous atrophy with chills. In the MEM and MEM + LFU groups, mice drank water occasionally without obvious shivering, and their mental state was better. The mice in the AMK and AMK + LFU groups had no obvious symptoms related to pneumonia, and their physical conditions almost recovered to a healthy state before infection.

### Real‐time fluorescence imaging and CPS quantification

**FIGURE 2:**
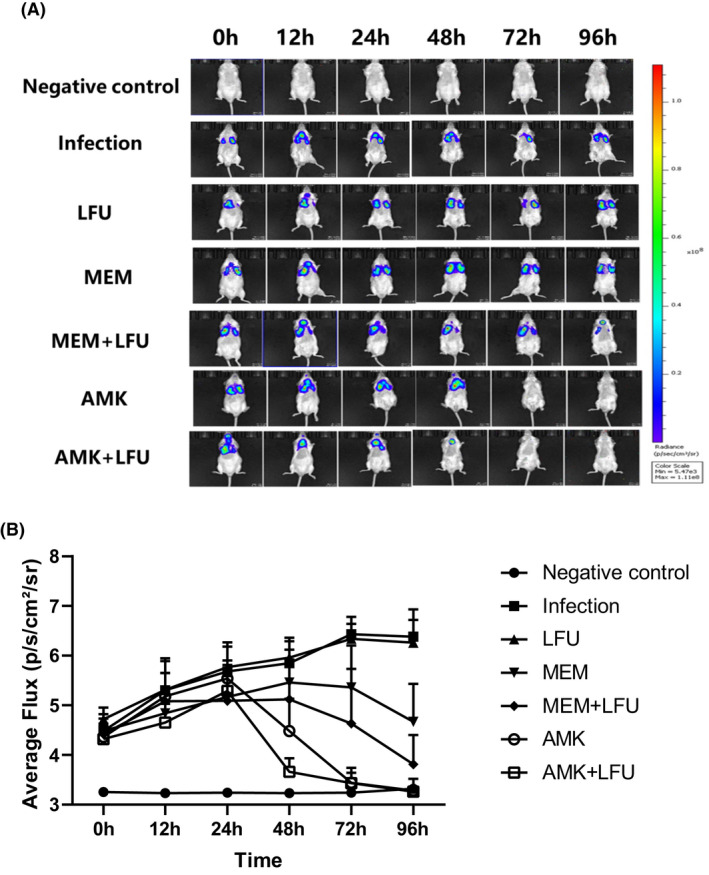
Representative bioluminescence monitoring images and CPS quantification analysis. (A) Representative bioluminescence monitoring images of Kp Xen39. The colour bar on the right represents the radiation intensity, and the brighter the colour, the more bacteria. (B) CPS quantification analysis and time‐varying trend of each group (means ± SD; *n* = 10).

The fluorescence intensity of all groups was gradually increased within 24 h of infection. From 24 to 96 h, the fluorescence intensity of the infection group and LFU group continued to increase, and that in the MEM and MEM + LFU groups was gradually decreased with time. At 96 h, the fluorescence intensity of the MEM + LFU group was gradually decreased, while that of the MEM group was not decreased obviously. The fluorescence intensity of the AMK and AMK + LFU groups was decreased from 24 h and disappeared completely at 72 h. The AMK + LFU group had a significantly lower fluorescence intensity at 48 h, while that of the AMK group was still relatively high (Figure 2A).

Figure 2B illustrates the quantitative analysis and the time‐varying trend of CPS. The average CPS of both the infection and LFU groups was continuously increased from 4.5 to 6.3 within 96 h. The average CPS of the MEM and MEM + LFU groups was increased to 5.5 and 5.1, respectively, at 48 h, and then it began to decline. At 96 h, the CPS of the MEM + LFU group was significantly lower than that in the MEM group (*p* < 0.05). The CPS of the AMK and AMK + LFU groups was increased to the highest level of 5.5 at 24 h. At 48 h, the CPS of the AMK + LFU group was decreased to 3.6, which was significantly lower than that in the AMK group with a CPS of 4.5 (*p* < 0.05). At 72 h, the CPS of AMK‐containing groups was almost reduced to the level of the negative control group.

### Lung bacterial load and relationship with CPS

**FIGURE 3:**
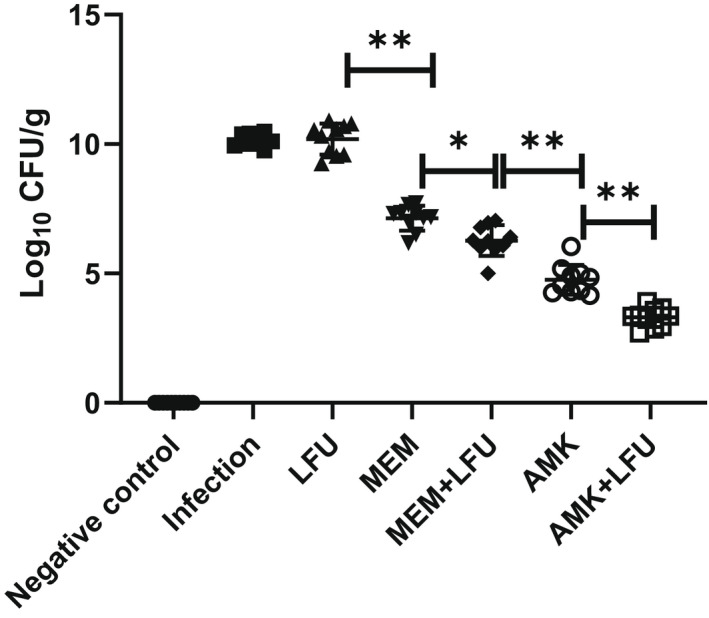
Total bacterial counts from the right lung homogenate of mice. (*n* = 10; *p < 0.05; **p < 0.01).

The tissue homogenate of the right lung was taken at 96 h to determine the bacterial load (Figure 3). The average CFU of the infection group and LFU group was up to 10.2 log_10_ CFU/g. The CFU of the antibiotics and antibiotics + LFU groups was significantly lower than that in the infection group. The CFU of the MEM and MEM + LFU groups was decreased to 7.1 and 6.2 log_10_ CFU/g, respectively. The CFU of the AMK group was decreased to 4.7 log_10_ CFU/g. The CFU of the AMK + LFU group was lower compared with the AMK group, reaching 3.2 log_10_ CFU/g.

### Lung histopathology and injury scores

**FIGURE 4:**
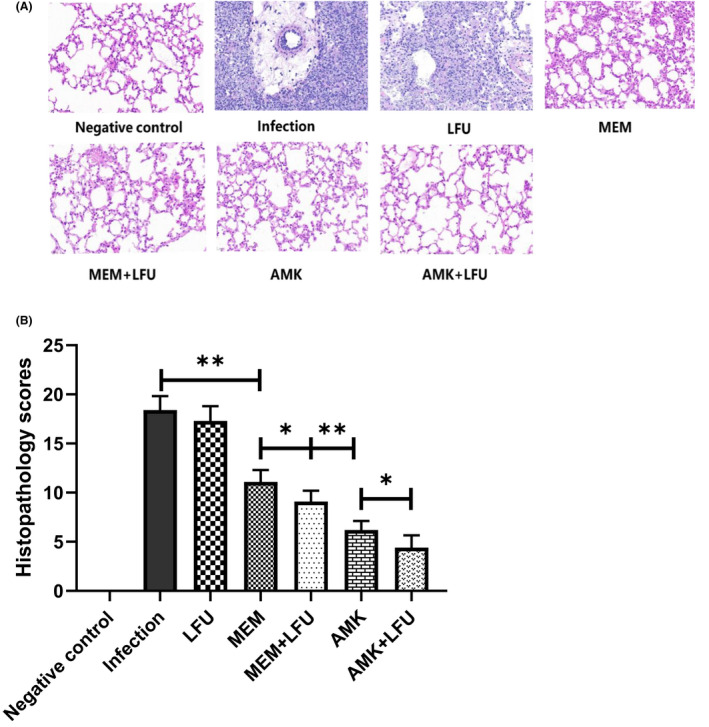
Lung histopathology and injury scores. (A) Representative H&E stained sections of mouse left lung. (B) Histopathological injure scores. (means ± SD; *n* = 10; *p < 0.05; **p < 0.01).

At 96 h, the left lungs of mice were stained with H&E and observed under a microscope (Figure 4A). Compared with the negative control group, the infection group and LFU group had obvious infiltration of inflammatory cells, large‐area necrosis of lung tissue, a large number of necrotic cell fragments, disintegration, destruction of the bronchial structure, and inflammatory cells in the bronchial lumen. The inflammation was gradually relieved in the antibiotics and antibiotics + LFU groups. Figure 4B shows that the average injury score of the infection group and LFU group was 18.4 and 17.3, respectively (*p* > 0.05). The scores of all four antibiotic groups were significantly lower than those in the infection group. AMK‐containing groups showed a greater degree of decline. The scores of the LFU combination groups were lower than those in the MEM or AMK alone group.

### Cytokine concentration in plasma and BALF

**FIGURE 5:**
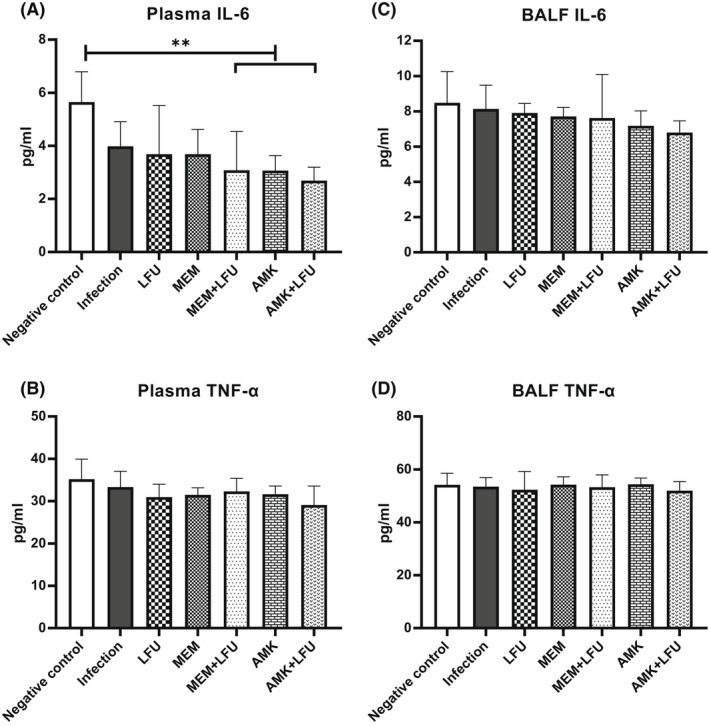
Cytokine concentrations (IL‐6 and TNF‐α) in plasma and BALF (means ± SD; *n* = 10; **p < 0.01).

The concentrations of IL‐6 and TNF‐α in plasma and BALF were measured at 96 h (Figure 5). Except that the concentration of IL‐6 in plasma of the MEM + LFU, AMK, and AMK + LFU groups was significantly lower compared with the negative control group (Figure 5A), and there was no statistical difference observed among other groups (Figure 5B–D).

### Antibiotic distribution in plasma and lung

**FIGURE 6:**
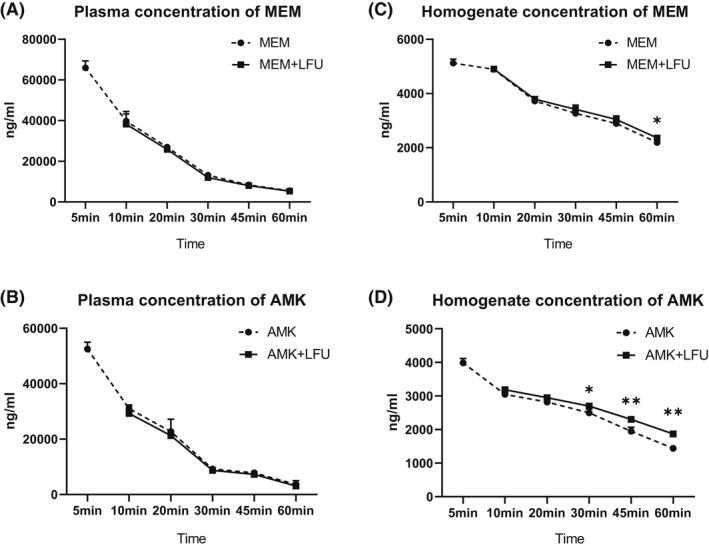
The concentration of antibiotics in plasm and homogenate (means ± SD; *n* = 6; *p < 0.05; **p < 0.01).

The concentrations of MEM and AMK in the plasma were similar between antibiotics and antibiotics + LFU groups (Figure 6A,B). At 60 min, the concentration of MEM in the lung tissue of the MEM + LFU group was higher than that of the MEM group (Figure 6C). The concentration of AMK in lung tissue was increased more obviously. From 30 min, the AMK concentration of the AMK + LFU group was higher than that of the AMK group. At 60 min, the difference in tissue concentration between the two groups reached 432 ng/ml (Figure 6D).

## DISCUSSION

In the present *in vivo* study, we provided evidence that LFU could improve the efficacy of MEM or AMK in the Kp pneumonia model. LFU in combination with antibiotics significantly improved the survival state of mice, decreased the CPS, accelerated the clearance of bacteria, and reduced inflammatory injury of the lung. As a safe and non‐invasive method, LFU can enhance tumour chemotherapy, gene therapy, and immunotherapy and increase the body's release of reactive oxygen species (ROS) (Hester et al., 2020). In the field of anti‐infection, an *in vitro* study has shown that LFU in combination with antibiotics can enhance the efficacy of antibiotics on planktonic bacteria and biofilm (Cai et al., 2017). In the rabbit and mouse implant models, LFU can enhance vancomycin and antimicrobial peptides in the treatment of Gram‐positive bacterial biofilm (Carmen et al., 2004; Li et al., 2015). In clinical application, LFU in combination with gentamicin solution can significantly reduce the incidence of septic complications in 17 patients (Komrakov & Antipov, 1962). The antibacterial efficacy of LFU in combination with chemotherapeutic drugs involves many mechanisms. The first and most important one is the cavitation effect of LFU. LFU stimulates the transport of antibiotics through cells and biofilms due to cavitation, high pressure, and high shear stress (Shi et al., 2013). Second, LFU can activate relevant sensitizing substances to achieve sonodynamic therapy and kill pathogens locally and accurately by releasing ROS (Juffermans et al., 2006). Third, LFU may also affect the microenvironment around pneumonia, induce immune activation, improve the release of inhibitory inflammatory factors, increase angiogenesis, and promote the repair of inflammation. Moreover, dormant bacteria in biofilm may be more sensitive to antibiotics after LFU treatment (Pitt et al., 1994). Generally, LFU increases the effectiveness of antibiotics by enhancing the rate of antibiotic transport to the bacteria, the permeability of the cell membrane, the metabolic activity of the bacteria, and oxygen and other nutrient transport.

The distribution of antibiotics in lung tissue revealed that LFU could promote the entry of MEM and AMK from blood vessels into lung tissue, especially for AMK with low distribution in lung tissue. This finding showed that LFU could not only promote medicine absorption through the epidermis but also enhance the penetration in the deep tissue of the body at a certain frequency and intensity. MEM and AMK are usually combined with cephalosporins in the treatment of Kp pneumonia, especially for drug‐resistant Kp (Ota et al., 2020). The reason may be attributed to that they are unevenly distributed in the body and the permeability of lung tissue is not adequate. A previous study has investigated the penetration of MEM in epithelial lining fluid (ELF) and alveolar cells (ACs), and the results have shown that the ELF/plasma penetration ratios range from 32% to 53%, and the AC/plasma penetration ratios range from 26% to 34% (Nicolau, 2008). Our data showed that under the LFU treatment, the ratio (lung/plasma) of MEM was slightly increased from 40% to 44% at 60 min. AMK exhibits concentration‐dependent bactericidal activity, which requires an adequate maximum concentration (*C*_max_)/MIC ratio for efficacy (Freeman et al., 1997). The latest global study shows that the sensitivity rate of Kp to AMK remains high at 83% (Wang et al., 2021). However, AMK is rarely selected as the first‐line treatment of pulmonary infection, which may be attributed to the poor permeability of AMK to the alveolar part of lung tissue (Honeybourne, 1994). In our present study, the ratio (lung/plasma) of AMK was increased from 39% to 60% at 60 min after LFU treatment. LFU increased the concentration of AMK in lung tissue, which greatly enhanced the bactericidal effect of AMK.

Furthermore, our findings were consistent with the previous results reported by Tian et al. (2020) showing that LFU promotes medicine penetration to improve the efficacy of intrapleural administration in the treatment of malignant pleural effusion. Similarly, some animal studies have also demonstrated the ability of LFU to promote drug release in implanted material (Cai et al., 2007; Yan et al., 2007). The mechanism of LFU promoting the distribution of antibiotics might involve not only the cavitation effect but also the thermal effect and acoustic microstreaming (Uddin et al., 2021). Because of its wave nature, LFU can be focused through tissue and directed to the desired target volume in the body. The energy generated by the rupture of cavitating vesicles will lead to the displacement of surrounding tissues, resulting in the disorder of lipid bilayer structure of cell membrane, which can increase the permeability of vascular endothelium and alveolar epithelium to improve the osmotic concentration of antibiotics (Azagury et al., 2014). The thermal effect produced by LFU leads to instantaneous increase of lung temperature, affects the fluidity of the phospholipid bilayer, and then impairs the permeability of cell membrane and vascular endothelium, resulting in increased permeability of antibiotics (Levenback et al., 2012). The acoustic microfluidic effect refers to the unidirectional liquid flow of LFU in the medium, which drives the transmission of antibiotics from a high concentration in blood vessels to a low concentration in alveoli (Baker et al., 2001).

Moreover, it has been reported that LFU can reduce inflammatory injury and promote tissue repair, including promoting fracture healing, accelerating tissue regeneration, and inhibiting inflammation (Mei & Zhang, 2021). This is consistent with our histopathological results that LFU reduced lung inflammatory injury in mice. These changes may be related to the upregulation of cell proliferation induced by LFU and the promotion of multidirectional differentiation of mesenchymal stem/progenitor cell lines through various signalling pathways (Kusuyama et al., 2014). LFU can enhance the proliferation and division of tissue cells and promote inflammatory repair (Breuing et al., 2005). In addition, LFU can increase the oxygen saturation at the inflammatory site, elevate the haemoglobin concentration, and improve the microcirculation at the inflammatory site (Wollina et al., 2011). IL‐6 is secreted by the white blood cells, such as T lymphocytes, macrophages, and endothelial cells. TNF‐α is mainly produced by activated monocytes–macrophages and various other cells. When lung inflammation occurs, the concentrations of IL‐6 and TNF‐a in serum and lung tissue are increased rapidly, which are typical cytokines in the body's inflammatory response. In our present study, the changing trend of IL‐6 and TNF‐α was not observed in plasma and BALF. However, Ho et al. (2020) have demonstrated that LFU can significantly regulate the changes of immune cells, cytokines, antigens, and antibodies in the tumour microenvironment, playing an important role in immunotherapy. The difference may be related to immunosuppression in mouse models applied in our study. Therefore, the effect of LFU on cytokines needs to be further evaluated in non‐immunosuppressive animals.

There are still some limitations of this study. First, we chose KpXen39 to facilitate real‐time observation of *in vivo* imaging, while it was a sensitive strain. The effects of LFU on pneumonia caused by resistant Kp and biofilm *in vivo* need to be further studied. Second, due to the strong ability of mice to eliminate pathogens, the mouse pneumonia model we built was immunosuppressed, which prevented us from investigating the effect of LFU on pulmonary inflammatory factors in mice. A non‐immunosuppressive mouse model of pneumonia needs to be established. Third, unfortunately, we did not find a statistically significant difference between the LFU and infection groups in the investigated indicators. However, in CPS and injury scores, the LFU group tended to be lower than the infection group. The reason for this lack of noticeable difference could be explained as follows. First, it might be related to the lower intensity and shorter duration of action of LFU. Second, the immunosuppressed mice masked the effects of LFU on regulating the body's immunity and microenvironment.

## CONCLUSIONS

As the first *in vivo* study of LFU in combination with antibiotics in the treatment of mouse pneumonia, we found that LFU in combination with MEM or AMK could significantly accelerate the clearance rate of pulmonary bacteria, reduce the inflammatory injury, and promote the distribution of antibiotics in lung tissue. The application of LFU in the treatment of pulmonary infection might have far‐reaching significance.

## AUTHOR CONTRIBUTIONS

Conception: Yun Cai; design of work: Kaicheng Yan; acquisition and analysis: Tianli Yang and Liuhan Dong; interpretation of data: Juan Xu and Jin Wang; drafting and revising of document: Kaicheng Yan and Yun Cai.

## FUNDING INFORMATION

This work was supported by the National Natural Science Foundations of China (81770004 and 82073894) and the Cultivation Project of PLA General Hospital for Distinguished Young Scientists (2020‐JQPY‐004).

## CONFLICT OF INTEREST

There is no conflict of interest to be declared. The authors alone are responsible for the content and writing of the paper.

## Data Availability

The data that support the findings of this study are available from the corresponding author upon reasonable request.
